# Critical roles of IL-6 signaling in myoblast differentiation of human adipose-derived mesenchymal stem cells

**DOI:** 10.1186/s41232-025-00373-6

**Published:** 2025-04-10

**Authors:** Takashi Otsuka, Kaoru Yamagata, Mai-Phuong Nguyen, Uyen Thi Ngo, Hidenori Sakai, Gulzhan Trimova, Junpei Anan, Yosuke Okada, Shingo Nakayamada, Yoshiya Tanaka

**Affiliations:** 1 The First Department of Internal Medicine, School of Medicine, University of Occupational and Environmental Health, Kitakyushu, Japan; 2 Department of Internal Medicine, High School of Medicine, Al-Farabi Kazakh National University, Al-Farabi Avenue 71, Almaty, 050040 Kazakhstan; 3 Oncology & Immunology Unit, Research Division, Mitsubishi Tanabe Pharma Corporation, 1000 Kamoshida, Aoba, Yokohama, Kanagawa Japan

**Keywords:** ADSCs, Myoblast, 5-aza-C, IL-6, STAT1

## Abstract

**Background:**

Ectopic fat is also formed in muscles as well as the liver, where adipose-derived mesenchymal stem cells (ADSCs) promote adipogenesis. On the other hand, after muscle injury, muscle satellite cells (SCs) contribute to muscle repair through myodifferentiation. Human ADSCs are multipotent stem cells, but it remains unclear whether they are involved in myoblast differentiation. The aim is to find a novel myogenic cytokine and its signaling pathway that promotes the differentiation of human ADSCs—a potential source of new muscle precursor cells—into myoblasts.

**Methods:**

An array kit was used to detect cytokines produced by ADSCs. After treating ADSCs with the DNA methyltransferase inhibitor 5-Aza-2’-deoxycytidine (5-aza-C) and different JAK inhibitors, MyHC1, a myodifferentiation marker, was detected by immunofluorescence staining and reverse transcription‐quantitative polymerase chain reaction (RT-qPCR). The expression status of signaling molecules was determined by Western blotting and the recruitment of transcription factors to the *MYOG* promoter by chromatin immunoprecipitation (ChIP).

**Results:**

IL-6 was detected at high concentrations in the culture supernatant of ADSCs. ADSCs stimulated with 5-aza-C became strongly positive for MyHC1 on day 21 post-stimulation. When co-stimulated with 5-aza-C and IL-6/sIL-6R, ADSCs became positive for MyHC1 protein and upregulated *MYOG* mRNA as early as day 14 post-stimulation. Co-stimulation with 5-aza-C and IL-6/sIL-6R resulted in phosphorylation of STAT1 and STAT3. The addition of a JAK2 inhibitor, but not JAK1/3 inhibitors, abolished the MyHC1 positivity and phosphorylation of STAT1 and STAT3. Co-stimulation with 5-aza-C and IL-6/sIL-6R during the myogenesis process resulted in the recruitment of STAT1, but not STAT3, to the *MYOG* promoter. Myoblast differentiation induced by stimulation with 5-aza-C was enhanced by activation of the IL-6/JAK2/STAT1/MYOG pathway.

**Conclusions:**

Therefore, sustained IL-6/JAK2/STAT1 activation may serve as an important driver of human ADSC differentiation into myoblast, suggesting an important candidate signaling pathway for ameliorating muscle atrophy.

**Supplementary Information:**

The online version contains supplementary material available at 10.1186/s41232-025-00373-6.

## Background

Muscle atrophy leads to a decline in quality of life (QOL) and activities of daily living (ADL), ultimately reducing healthy life expectancy [1]. Muscle atrophy is a common condition not only in patients with muscle injuries but also in healthy individuals. Age-related changes in body composition are characterized by reduced skeletal muscle mass and strength, a condition known as sarcopenia [2, 3]. It is associated with frailty, which is a clinical syndrome of age-related decline in physiologic reserve and increased vulnerability [4]. However, since there are no effective therapeutic drugs, basic research is essential to elucidate the triggers of muscle differentiation and the related signaling pathways. In particular, the discovery of progenitor cell sources of myofibers and the in vitro establishment of myodifferentiation systems will be beneficial for the future in developing strategies to maintain skeletal muscle mass and strength.

A population of stem cells called satellite cells (SCs), which are localized in the niche beneath the basement membrane of muscle fibers, are rapidly activated in response to local acute tissue injury to proliferate and differentiate into myoblasts, which are fused to repair the injured muscle fibers [5, 6]. Thus, these SCs with muscle-regenerating capabilities are important for maintaining muscle homeostasis. In contrast, cell surface molecules CD73, CD90, CD105, CD146, and human leukocyte antigen (HLA)-ABC, which are expressed on mesenchymal stem cells (MSCs), are also expressed on myoblasts [7]. Interestingly, when stimulated with galectin-1 (LGALS1), a β-galactoside-binding protein secreted by myoblasts, human fetal MSCs formed multinucleated muscle fibers, and expressed the muscle markers DESMIN, myogenic transcription factors such as myoblast determination protein 1 (MYOD1) and myogenin (MYOG) [8]. Additionally, 5-Aza-2’-deoxycytidine (5-aza-C), a DNMT1 inhibitor, hypomethylated DNA residues and activated the muscle-specific intermediate filament protein DES in muscle progenitor cells, leading to myogenesis [9].

On the other hand, muscles contain adipose-derived MSCs (ADSCs) as well as SCs because of the presence of fat, and murine ADSCs can differentiate into muscle cells [10]. Given that patients with sarcopenia have a decreased number and function of SCs [11], we focused on ADSCs in this study. In vitro, human ADSCs can differentiate into osteoblasts, adipocytes, and chondrocytes [12–14]. However, it remains unclear whether human ADSCs can differentiate into myoblasts in vitro.

Myogenesis is a complex process following multiple differentiation steps, involving MYOD and MYOG, cytoskeletal proteins such as intermediate filaments DESMIN and myosin heavy chain (MyHC). MYOD is regulated by DNA methylation and induces the gene expression of MYOG, a myodifferentiation transcription factor [15], which induces the expression of most muscle-specific genes [16]. DES forms the sarcoplasmic reticulum network [17]. Adult skeletal muscle fibers are classified into skeletal sarcomeric MyHC I, IIa, IIx, and IIb [18]. In general, MyHC I and MyHC IIa are predominantly expressed in slow oxidative muscle fibers, whereas MyHC IIb is predominantly expressed in fast glycolytic muscle fibers [18].

Myokines are cytokines produced by muscles, and IL-6 is a typical example. Under physiological conditions, IL-6 is produced locally and transiently by skeletal muscles and plays an important role in the maintenance of muscle homeostasis [19]. IL-6 levels in circulation are usually very low or undetectable but can rise dramatically in several pathological conditions [20]. In C2C12 cells, the knockdown of *STAT3* and *IL-6* mRNAs decreases the expression of MYOG and MyHC IIb, thereby inhibiting myotube fusion [21]. Similarly, other cytokines such as IL-1β and TNF-α act on C2C12 cells to inhibit myodifferentiation [22]. However, their effects on human ADSCs and myogenesis are yet to be investigated.

This study aimed to identify factors that control the efficient differentiation of human ADSCs into myoblast and elucidate their mechanisms of action.

## Methods

### Cell culture

Five distinct lots of human adipose-derived MSCs (ADSCs; see Table 1 for the detailed information) were purchased from Lonza (PT-5006; MD, USA). For these ADSCs, cell surface markers, including CD29, CD44, CD105, and CD166, were positive, while CD14, CD34, and CD45 were negative as indicated by the manufacturer. ADSCs were cultured as described previously [12]. Briefly, the ADSCs were cultured at 37 °C under a 5% CO_2_ atmosphere with a MSC growth medium (MSCGM; PT-3001; MGM Bullet Kit; Lonza) in 75 cm^2^ flask (2–8589-02, AS ONE) and their confluence was maintained at < 80% to prevent spontaneous differentiation. For cell passaging, ADSCs were detached from the flask by treating with trypsin at 37 °C for 3 min, followed by dilution in MSCGM to stop the reaction. For cryopreservation, after centrifugation at room temperature at 2000 rpm, the ADSCs pellet was suspended in 1 ml of cell cryopreservation solution (CELLBANKER, CB011, TaKaRa) and transferred into cryovials (430,489, CORNING) for storage in a liquid nitrogen tank. For thawing the cells, the cryovials containing ADSCs were retrieved from the liquid nitrogen tank and quickly thawed by placing them in a 37 °C water bath for 2 min. Immediately after thawing, MSCGM was added to dilute the cryopreservation solution, and the cells were seeded in a flask with MSCGM and cultured. All experiments were conducted using ADSCs at passage P3-P4. In contrast, human skeletal muscle myoblasts (HSMMs) were sourced from Lonza (CC-2580) and cultured at 37 °C under a 5% CO_2_ atmosphere with SkGM™−2 Skeletal Muscle Cell Growth Medium-2 BulletKit™ (Lonza; CC-3245) for 2 days.

**Table 1:** ADSC metadata

Unique ID	Age	Sex	Race	Adhesion ratio (%)	Survival rate (%)	Doubling time (H)
PT-5006 0000439845	65 Y	Female	Caucasian	64	89	26
PT-5006 0000440549	44 Y	Female	African American	58	72.6	22
PT-5006 0000535975	33 Y	Male	African American	47	87	24
PT-5006 0000543183	30 Y	Female	Asian/Orienntal	70	81	19
PT-5006 0000547705	89 Y	Male	Caucasian	72	91	28.8

### 5-aza-C treatment

ADSCs were cultured with Dulbecco’s modified Eagle medium (DMEM) containing 10% (v/v) fetal bovine serum (BioWest, Nuaille, France) and 1% penicillin–streptomycin and stimulated with 5-aza-C (1, 10 μM) at every 3 days for further analyses as described previously [23].

### Reagents

The detailed information in the present study is listed in Supplementary Table S1 (Additional file 8) and S2 (Additional file 9).

### Immunofluorescence staining

Immunofluorescence staining was performed as described previously [24, 25]. Briefly, the cells were immersed in phosphate-buffered saline with Tween (PBST; 0.05% [v/v], pH 6.0) containing sodium citrate (5 mM) to inactivate antigens. The samples were blocked with a serum-free protein block (Dako, #2016–08) and reacted with polyclonal antibodies that recognize human MyHC1 and 7 (dilution, 1:200; Abcam, Cambridge, MA). The slides were rinsed with PBST and then incubated for 1 h with an anti-rabbit IgG secondary antibody labeled with fluorescein isothiocyanate and an anti-mouse IgG secondary antibody labeled with rhodamine (dilution, 1:500; DakoCytomation, Glostrup, Denmark). Nuclei were stained with 4’, 6-diamidino-2-phenylindole (DAPI; dilution, 1:200; Merck). The stained samples were examined under an all-in-one fluorescence microscope.

### Cytokine array

To detect the secretion of cytokines in MSCGM (TaKaRa, C-28009), a human cytokine antibody array (Abcam, Cambridge, UK, Ab133998) was carried out. The culture supernatant was prepared by plating the cells on a 6-well plate at a density of 20,000 cells per cm^2^ and grown for 2 days. Two days prior to collecting the samples, the medium was replaced with 5 mL of fresh medium. The collected medium was filtered through a 0.22-µm filter and stored at − 80 °C. For the analysis, media were pooled and array performed with undiluted medium according to the manufacturer’s instructions. Chemiluminescent detection was performed with a ChemiDoc imaging system (Bio-Rad Finland, Helsinki, Finland), and densitometric data was obtained using ImageJ software [26]. Mean intensities of negative and positive control spots were used for background correction and normalization, respectively.

### Measurement of IL-6 in culture supernatants

Culture supernatants were collected 2 days after the last medium change at the indicated time points and stored at − 80 °C until analyzed. IL-6 concentrations were measured by cytometric bead array (Becton Dickinson) using a FACSVerse flow cytometer (Becton Dickinson) as described previously [27, 28], and data were analyzed using the FCAP Array software (BectonDickinson).

### Reverse transcription-quantitative polymerase chain reaction (RT-qPCR)

RNA extraction and RT-qPCR were performed as described previously [28, 29]. Briefly, cultured cells were lysed in RLT buffer purchased from Qiagen (Hilden, Germany). The total RNA was extracted using the RNeasy Mini Kit (Qiagen). For the total RNA, complementary DNAs (cDNAs) were synthesized using a high-capacity cDNA reverse transcription kit (Thermo Fisher) in accordance with the manufacturer’s instructions. Real-time PCR was performed in a StepOnePlus system (Applied Biosystems, Thermo Fisher). The relative quantities of the transcripts were analyzed using the 2 − △△Ct method and normalized to the amounts of *glyceraldehyde-3-phosphate dehydrogenase* (*GAPDH*). PCR was performed using specific primers (Supplementary Table 2; Additional file 9).

### Western blotting

Western blotting was carried out as described previously [24, 30]. Briefly, ADSC cells at indicated time points were sonicated (on/off cycle time: 30 s/30 s; cycle number: 5) in TNE lysis buffer containing 50 mM Tris (pH 8.0), 150 mM NaCl, and 1% Nonidet P40, supplemented with protease inhibitor cocktail (A-0014–20, ITSI Biosciences) and centrifuged at 12,000 × g for 30 min at 4 °C. The supernatant obtained after the centrifugation was used as whole-cell lysates (WCLs). WCL proteins (10 μg) were separated by 4–20% SDS-PAGE Tris–glycine gel and transferred onto a 0.2-μm nitrocellulose membrane (GE Healthcare). Immunoblotting was performed with primary antibodies followed by the appropriate secondary antibodies. β-actin was used as the loading control.

### Chromatin immunoprecipitation-PCR (ChIP-PCR)

ChIP-PCR was performed as described previously [27]. Human ADSCs were cultured with 5-aza-C for 5 days, starved for 1 day and then stimulated with or without 5-aza-C + IL-6/sIL-6R for 20 min. Chromatin was crosslinked with 1% formaldehyde for 10 min and fragmented to 200–500 bp by sonication. DNA was extracted using the EZ-ChIP Kit (Merck Millipore, Taufkirchen, Germany) using the protocol provided by the manufacturer. DNA was immunoprecipitated using a series of antibodies. PCR was performed using specific primers (Supplementary Table 2; Additional file 9).

### Statistical analysis

All quantified data are expressed as mean ± standard deviation. Differences between the two groups were tested for statistical significance by the Student’s unpaired two-tailed *t* test or Dunnett’s multiple comparison test. The statistical significance was set at *P* < 0.05. All statistical analyses were performed using SPSS statistical software (v. 21.0; IBM Corp., Armonk, NY).

## Results

### Human ADSCs produce IL-6 at high levels

**Fig. 1:**
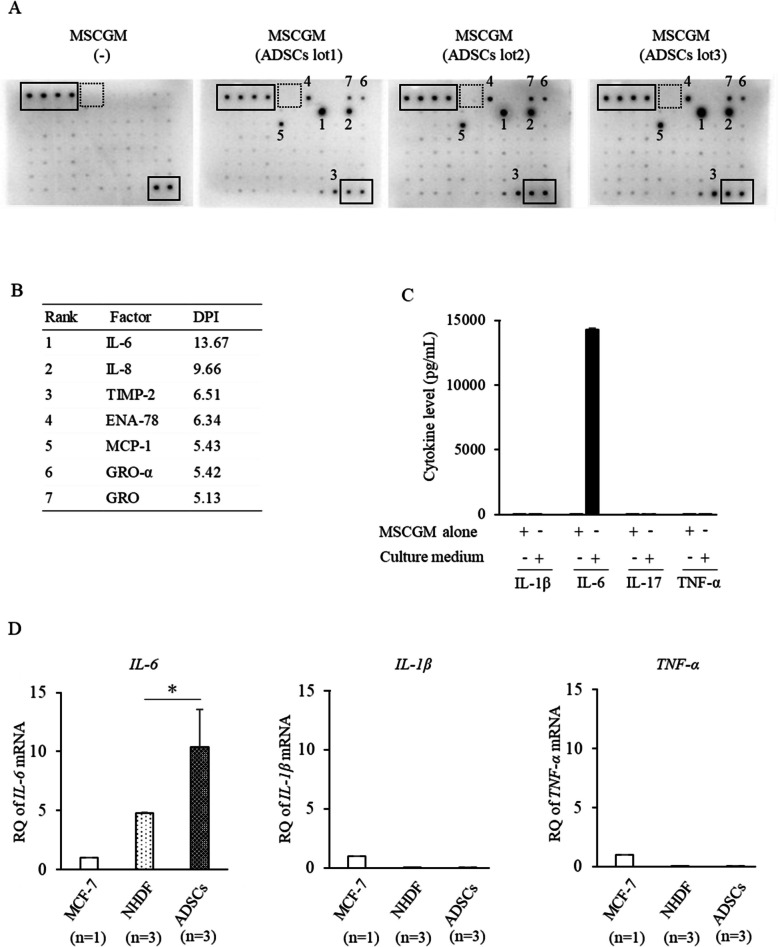
Human ADSCs produce IL-6 at high levels. **A** Cytokine array with ADSC culture supernatant. The one on the left was with MSCGM alone, and the three on the right were with ADSC culture supernatant. The solid and dashed frames indicate the positive and negative internal controls, respectively. Cytokines produced at significant levels were numbered 1 to 7. Three independent lots of ADSCs were used. **B** The top 7 factors with high secretion levels. DPI, dots per inch. **C** A panel of cytokines was measured in the culture supernatant of ADSCs using the CBA method. **D** The production of *IL-6*, *IL-1β*, and *TNF-α* from human breast cancer cell line (MCF-7), normal human dermal fibroblasts (NHDF), and three lots of ADSCs was quantified by RT-qPCR. **C**, **D** Three independent lots of ADSCs (**C**, **D**) and NHDF (**D**) were used. Data are expressed as mean ± standard deviation. Student’s unpaired two-tailed *t*-test was used for comparisons between two groups

Human ADSCs (#1, #2, #3, #4, and #5) were cultured for 48 h in a mesenchymal stem cell growth medium (MSCGM), and cytokines, chemokines, and growth factors in the culture supernatant were detected using a commercially available cytokine assay kit (Fig. 1A, and Supplementary Table 3; Additional file 10). As shown in the table of the top seven factors with high production levels, IL-6 showed the highest level of production (Fig. 1B). When quantified by the cytometric bead array (CBA) method, IL-6 was detected at a high level, whereas IL-1β, IL-17, and TNF-α were hardly detected (Fig. 1C). Among the human breast cancer cell line (MCF-7), human dermal fibroblasts (NHDF), and human ADSCs, ADSCs showed the highest expression of IL-6 by quantitative PCR (Fig. 1D). In contrast, IL-1β and TNF-α were highly expressed in MCF-7 cells but hardly detected in ADSCs (Fig. 1D).

### IL-6/sIL-6R promotes myoblast differentiation induced by 5-aza-C

**Fig. 2:**
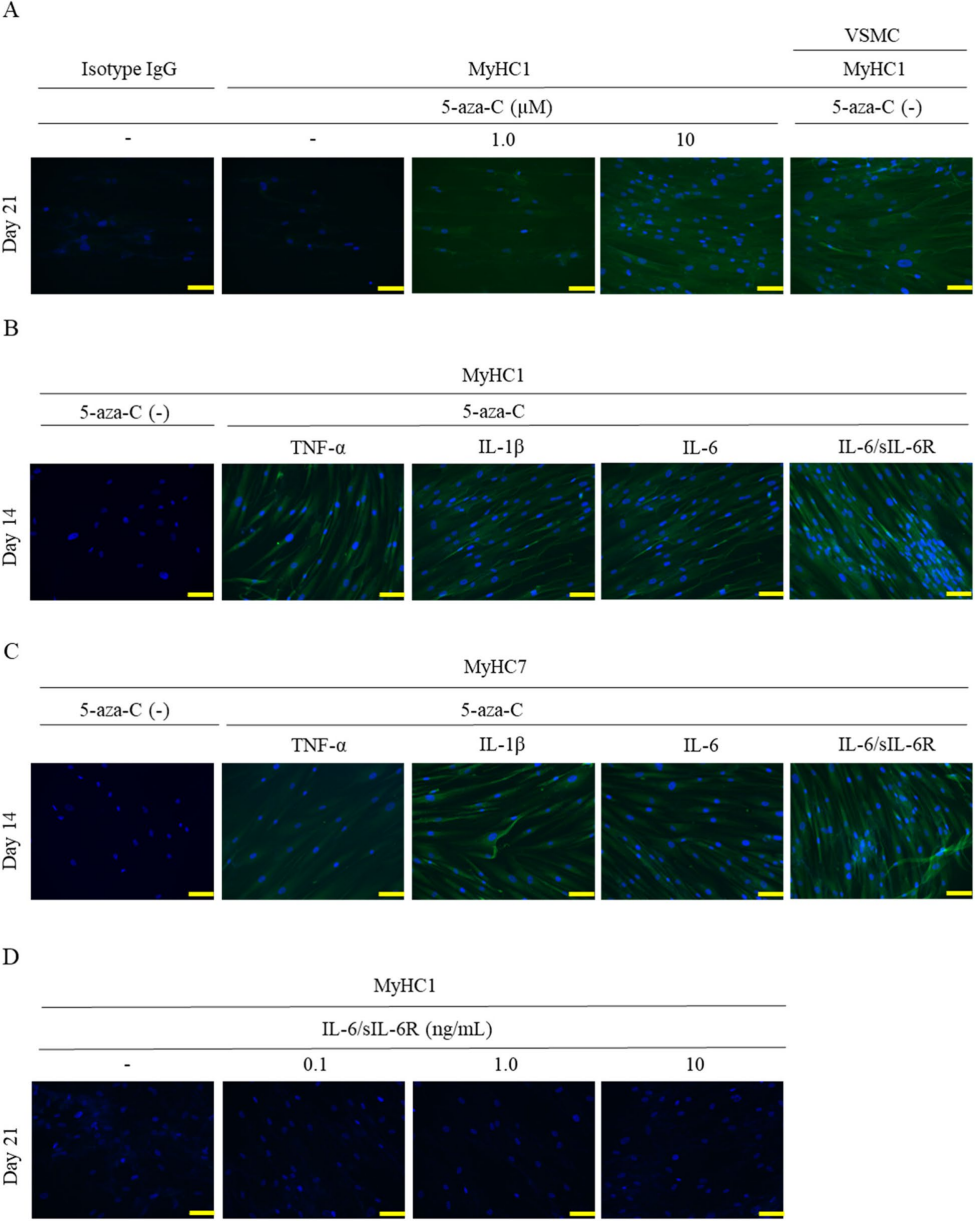
IL-6/sIL-6R promotes myoblast differentiation induced by 5-aza-C.** A**–**D** ADSCs were stimulated with 5-aza-C alone (1.0, 10 μM) (**A**) or co-stimulated with 5-aza-C (10 μM) and TNF-α (10 ng/mL), IL-1β (10 ng/mL), IL-6 (10 ng/mL), or IL-6/sIL-6R (10 ng/mL) (**B**,** C**), or varying concentrations of IL-6/sIL-6R (0.1, 1.0, 10 ng/mL) (**D**), followed by fluorescence staining with anti-MyHC1 antibody on days 14 (**B**) and 21 (**A**, **D**). Cells were also fluorescence-stained with anti-MyHC7 antibody on day 21 (**C**). Human vascular smooth muscle cells (VSMCs) were used as the positive control. Three independent lots of ADSCs were used. Data from a representative lot are presented. The images were captured at 200 × magnification. Scale bar indicates 100 μM

When stimulated with 5-aza-C alone, ADSCs became strongly positive for the fast muscle marker MyHC1 on day 21 post-stimulation in a concentration-dependent manner (1.0 µM, 10 µM) (Fig. 2A). Positive control human vascular smooth muscle cells (VSMC) were strongly positive for MyHC1. When ADSCs were co-stimulated with 5-aza-C (10 µM), TNF-α (10 ng/mL), IL-1β (10 ng/mL), IL-6 (10 ng/mL), or IL-6/sIL-6R (10 ng/mL), only IL-6/sIL-6R-stimulated cells became strongly positive for MyHC1 and MyHC7 on day 14 post-stimulation (Fig. 2B,C). When incubated with IL-6/sIL-6R (0.1, 1.0, and 10 ng/mL) in the absence of 5-aza-C, ADSCs remained negative for MyHC1 at all concentrations until day 21 post-stimulation (Fig. 2D).

### Co-stimulation of human ADSCs with 5-aza-C and IL-6/sIL-6R induces expression of skeletal muscle lineage cell markers

**Fig. 3:**
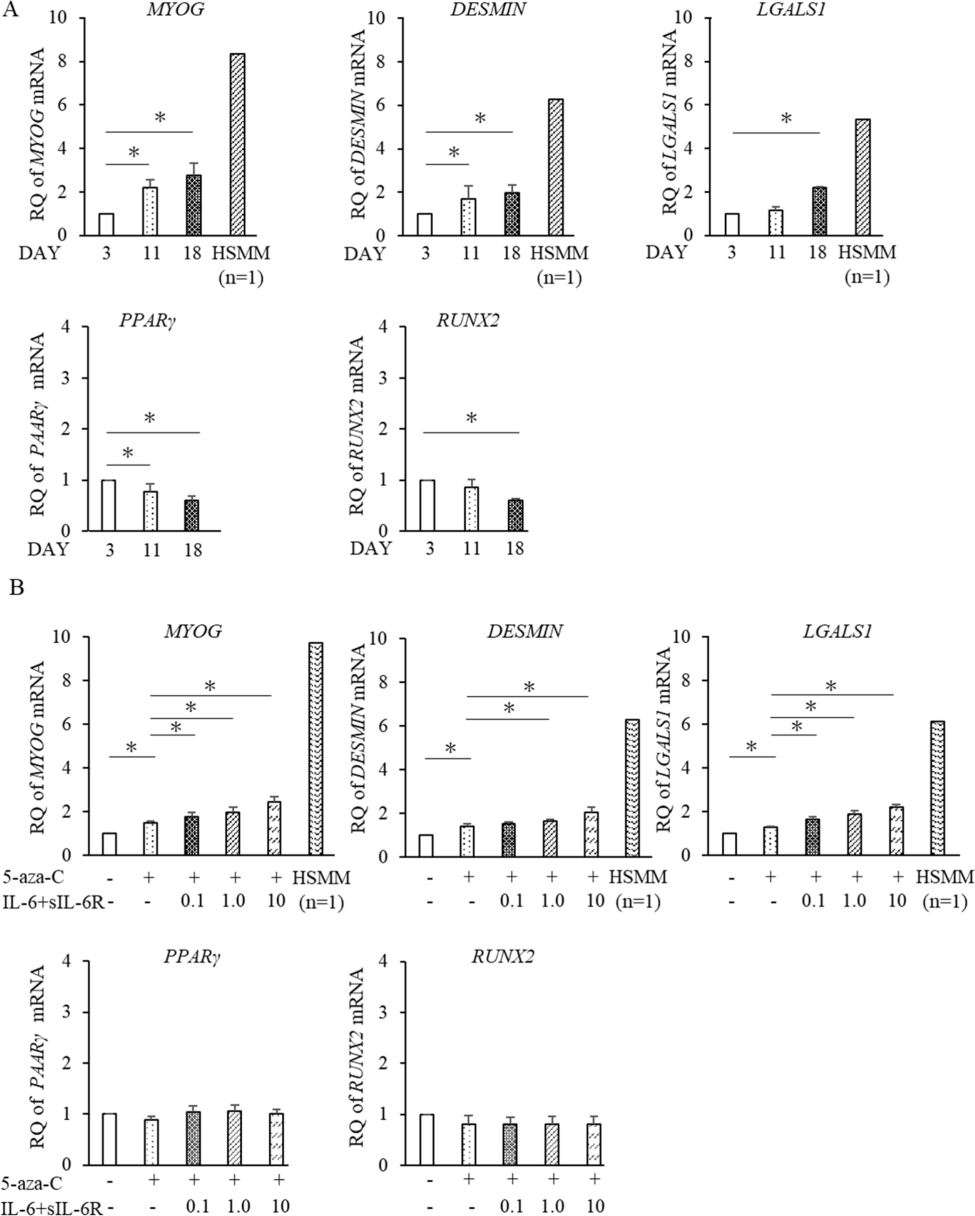
Co-stimulation of human ADSCs with 5-aza-C and IL-6/sIL-6R induces expression of skeletal muscle lineage cell markers. **A**, **B** ADSCs were stimulated with 5-aza-C alone (**A**) or co-stimulated with 5-aza-C and different concentrations of IL-6/sIL-6R (**A**, **B**). The expression of *MYOG*, *DESMIN*, *LGALS*, *PPARγ*, and *RUNX2* was quantified by RT-qPCR. Human skeletal muscle myoblasts (HSMMs) were used as the positive control. Five independent lots of ADSCs were used. Data are expressed as mean ± standard deviation. Student’s unpaired two-tailed *t*-test was used for comparisons between two groups

Stimulation with 5-aza-C elicited *MYOG*, *DESMIN*, and *Lectin Galactoside-Binding Soluble 1* (*LGALS1*) gene expression in ADSCs over time (days 3, 11, and 18), whereas *PPARγ* and *RUNX2* expressions declined (Fig. 3A). In contrast, the expression levels of *MYOG*, *DESMIN*, and *LGALS1* mRNAs were lower in ADSCs stimulated with 5-aza-C for 18 days compared to human skeletal muscle myoblasts (HSMMs) (Fig. 3A). IL-6 production gradually decreased on days 0, 7, 14, and 21 after stimulation with 5-aza-C (Supplementary Figure S1). When co-stimulated with 5-aza-C and IL-6/sIL-6R for 11 days, ADSCs showed increased expression of the *MYOG*, *DESMIN*, and *LGALS1* in an IL-6/sIL-6R concentration-dependent manner (0.1, 1.0, and 10 ng/mL), with unchanged expression of the *PPARγ* and *RUNX2* genes (Fig. 3B). In contrast, the expression level of DESMIN protein was lower in ADSCs stimulated with 5-aza-C for 14 days compared to HSMMs (Supplementary Figure S2 and Additional file 1). Following stimulation with TNF-α (Supplementary Figure S3A) or IL-1β (Supplementary Figure S3B), there was no change in the expression of the *MYOG*, *PPARγ*, or *RUNX2* gene, regardless of the concentration (0.1, 1.0, or 10 ng/mL). Co-stimulation with IL-6/sIL-6R in the absence of 5-aza-C resulted in unchanged expression of the *MYOG*, *PPARγ*, or *RUNX2* gene, regardless of the concentration of IL-6/sIL-6R (0.1, 1.0, or 10 ng/mL) (Supplementary Figure S4).

### Stimulation of human ADSCs with IL-6/sIL-6R induces phosphorylation of STAT1/3, which is inhibited by a JAK2 inhibitor

**Fig. 4:**
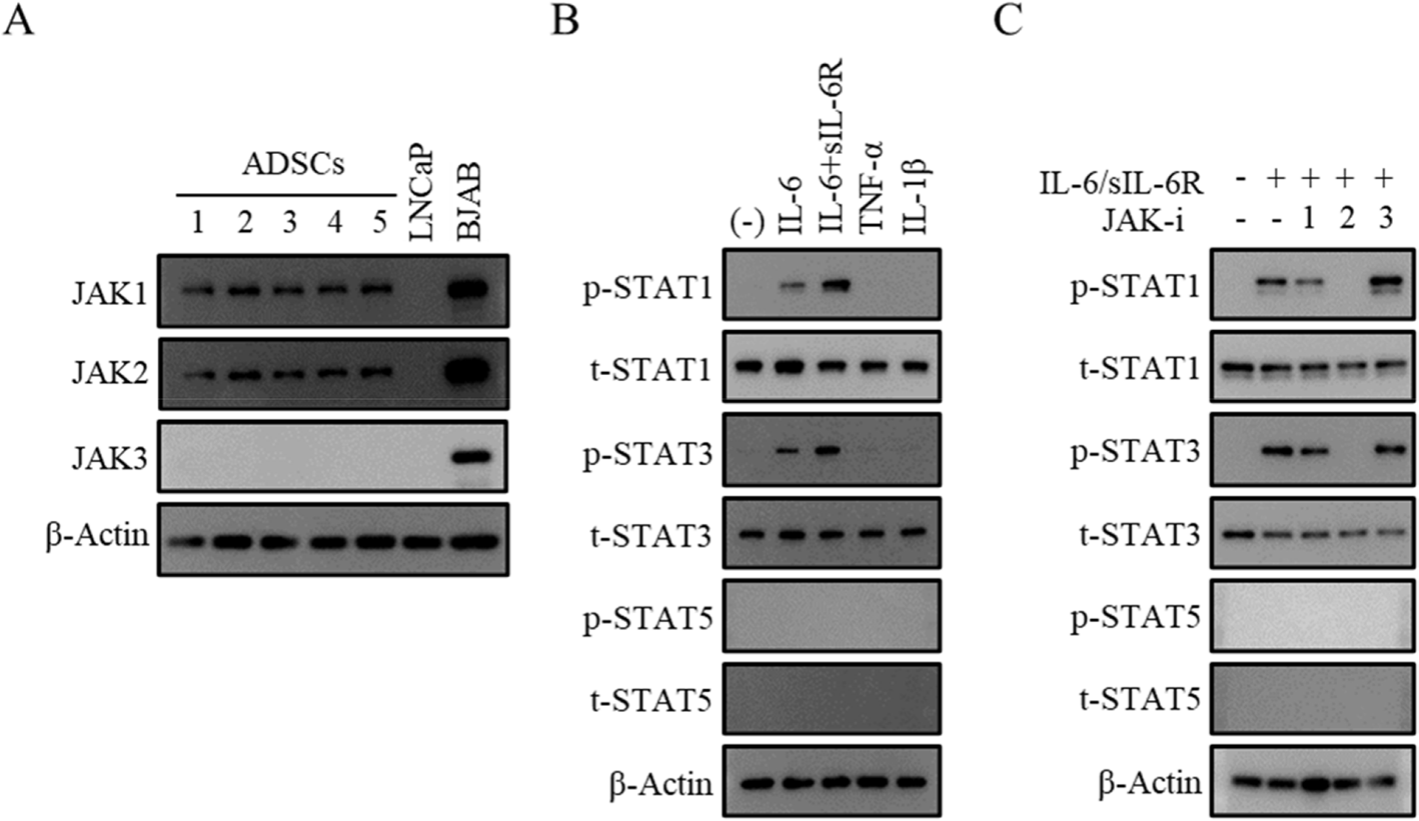
Stimulation of human ADSCs with IL-6/sIL-6R induces phosphorylation of STAT1/3, which is inhibited by a JAK2 inhibitor. **A**–**C** The whole-cell extract of ADSCs (lot #1, 2, 3, 4, and 5) was subjected to Western blotting (WB). **A** The expression of JAK1, JAK2, and JAK3 molecules was examined. BJAB and LNCaP cells were used as controls. **B** Following co-stimulation with 5-aza-C and IL-6, IL-6/sIL-6R, TNF-α, or IL-1β (30 min), expression of STAT1 and STAT3 was determined. **C** Cells were pre-treated with a JAK1, JAK2, or JAK3 inhibitor for 1 h, stimulated with IL-6/sIL-6R for 30 min, and then measured for STAT1, STAT3, and STAT5 expressions. Five independent lots of ADSCs were used. Data from a representative lot are presented

When the whole-cell extract of ADSCs was subjected to Western blotting, JAK1, 2, and 3 proteins were detected in ADSCs (Fig. 4A and Additional file 2). The human Burkitt’s lymphoma B cell line BJAB and the human prostate cancer cell line LNCaP were used as controls. ADSCs that had been serum-starved for 24 h were stimulated with a series of cytokines for 20 min. The results showed that phosphorylation of STAT1 and STAT3 proteins was induced most strongly after co-stimulation with IL-6/sIL-6R (Fig. 4B and Additional file 3). STAT5 was not detected in any of the samples (Fig. 4B and Additional file 4). ADSCs were then treated with each of the different JAK inhibitors for 1 h, stimulated with IL-6 + s-IL-6R for 30 min, and then subjected to the detection of different STAT proteins. Stimulation of ADSCs with a JAK2-specific inhibitor, but not with a JAK1- or JAK3-specific inhibitor, resulted in suppressed phosphorylation of STAT1 and STAT3 (Fig. 4C and Additional file 5). STAT5 was not detected in any of the samples (Fig. 4C and Additional file 6).

### Stimulation with a JAK2 inhibitor inhibits myogenesis of human ADSCs

**Fig. 5:**
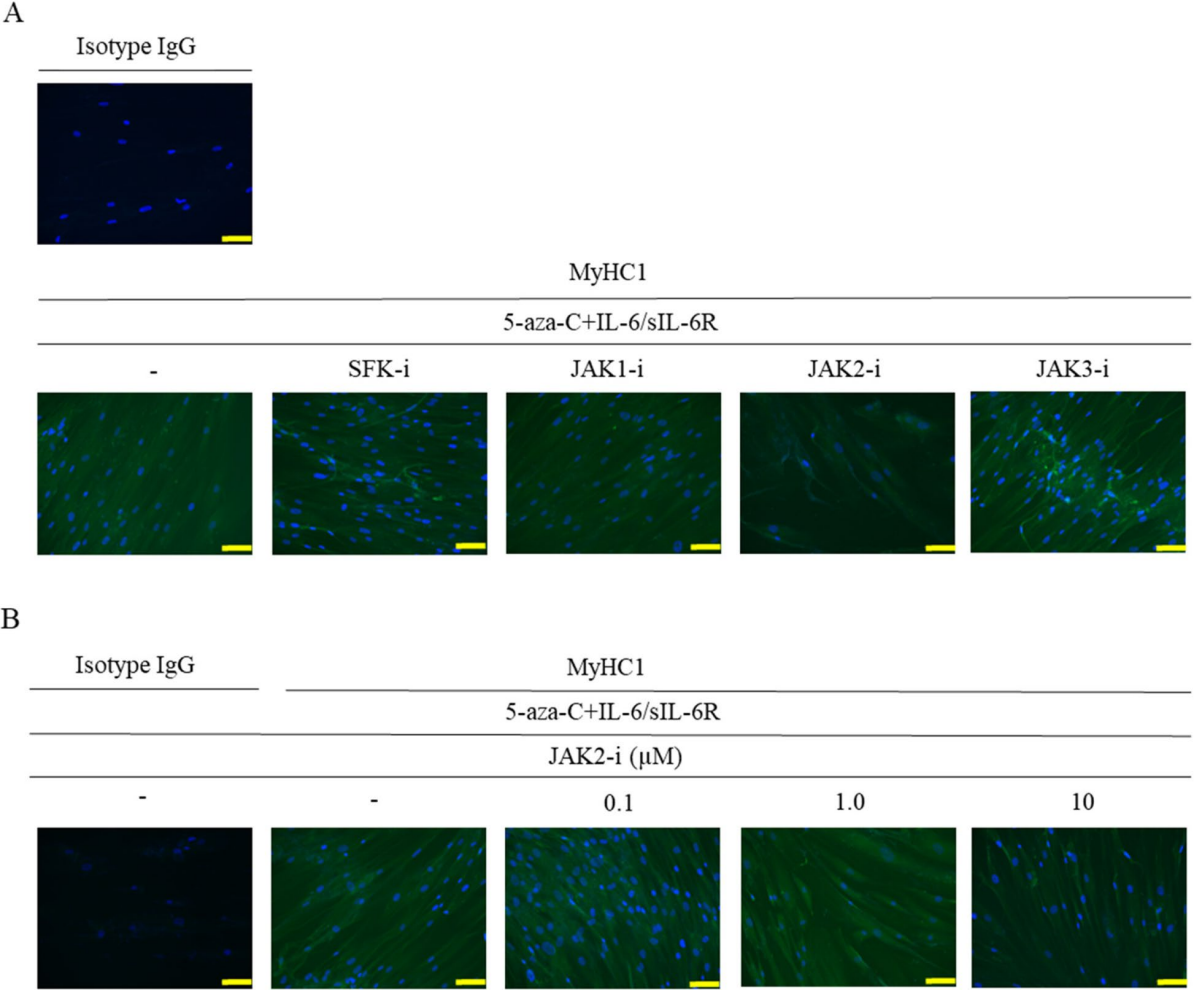
Stimulation with a JAK2 inhibitor inhibits myogenesis of human ADSCs. **A**, **B** When co-stimulated with 5-aza-C and IL-6/sIL-6R, ADSCs became strongly positive for MyHC1 on day 21 post-stimulation. **A** Cells were pre-treated with a SFK, JAK1, JAK2, or JAK3 inhibitor (10 μM) for 1 h. **B** Cells pre-treated with a JAK2 inhibitor at concentrations of 0.1, 1, and 10 μM were stained for MyHC1. Representative photographs from three independent experiments that showed similar findings are shown. The images were captured at 200 × magnification. Scale bar indicates 100 μM

When co-stimulated with 5-aza-C and IL-6/sIL-6R, ADSCs became strongly positive for MyHC1 on day 14 post-stimulation, which was maintained even after treatment with inhibitors of SFK, JAK1, and JAK3. However, the cells were only weakly stained for MyHC1 when treated with a JAK2 inhibitor (Fig. 5A). Moreover, when ADSCs were pre-treated with a JAK2 inhibitor for 1 h, subsequent co-stimulation with 5-aza-C and IL-6/sIL-6R resulted in a decreased positivity for MyHC1 in a JAK2 inhibitor concentration-dependent manner (Fig. 5B).

### Co-stimulation of ADSCs with IL-6/sIL-6R induced recruitment of STAT1 to the MYOG gene promoter

**Fig. 6:**
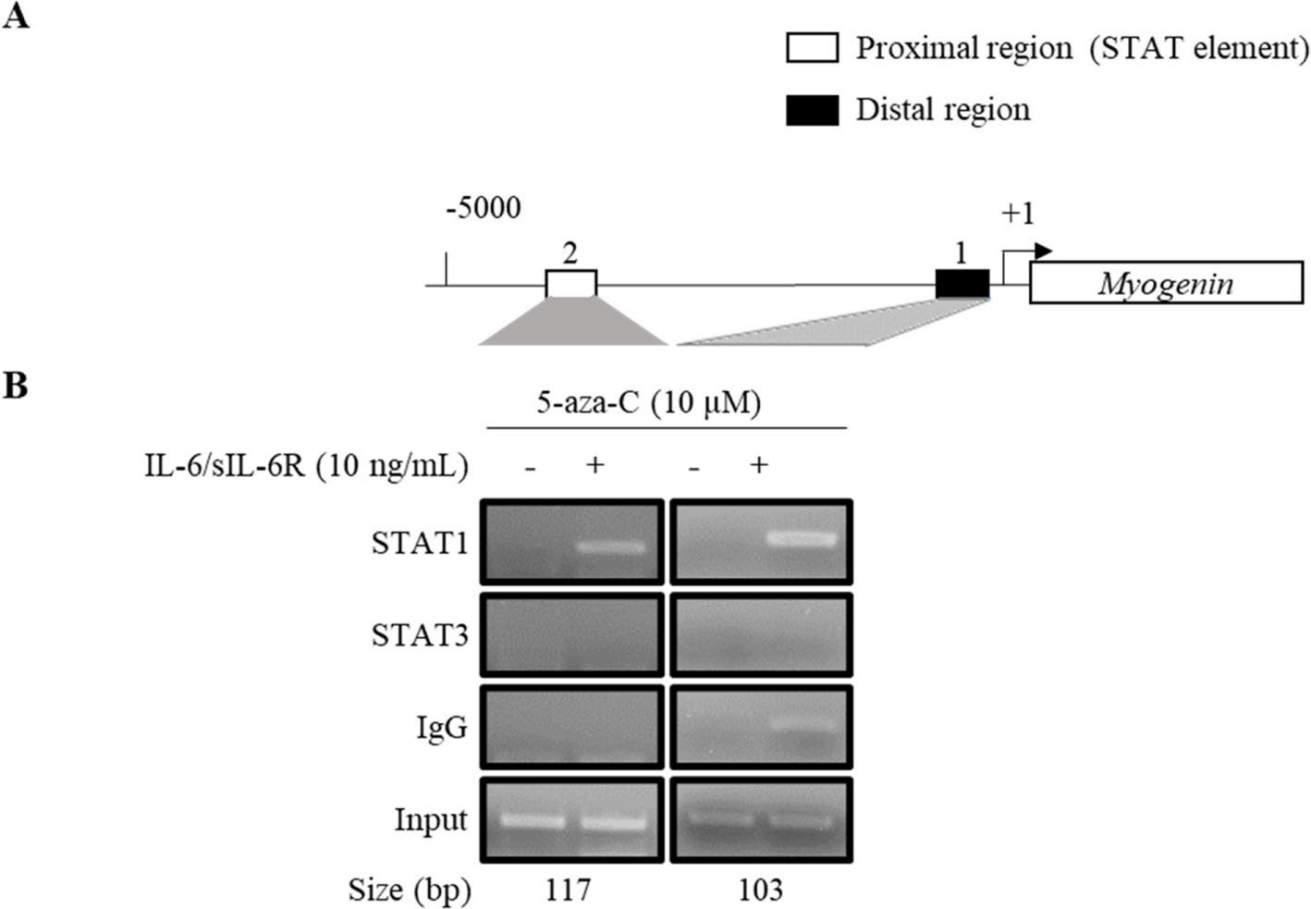
Co-stimulation of ADSCs with IL-6/sIL-6R induced recruitment of STAT1 to the *MYOG* gene promoter. **A** Expected STAT binding site of the *MYOG* gene promoter.** B** ADSCs incubated with or without 5-azaC and IL-6/sIL-6R (30 min) were subjected to ChIP assay using a series of antibodies

An expected STAT-binding site was found in the promoter region of the *MYOG* gene (Fig. 6A). At 24 h after co-stimulation with 5-aza-C and IL-6/sIL-6R, STAT1, but not STAT3, was recruited to the *MYOG* promoter (Fig. 6B and Additional file 7). Meanwhile, none of the STAT molecules was recruited in the distal region.

## Discussion

ADSCs were cultured after stimulation with 5-aza-C and IL-6/sIL-6R. The cells became strongly positive for MyHC1 and differentiated into myogenic lineage cells on day 14 post-stimulation. Myodifferentiation was inhibited by JAK2 inhibition, demonstrating the role of JAK2 activation in myodifferentiation. Furthermore, STAT1 was recruited at the STAT-binding site in the proximal region of the promoter of *MYOG*, a transcription factor regulating myodifferentiation. These findings indicate that activation of the IL-6/JAK2/STAT1/MYOG pathway plays a role in the myodifferentiation of ADSCs in vitro.

ADSCs localized in adipose tissues have been shown to differentiate into osteoblasts and adipocytes in in vitro systems [12, 13]. One of the important findings of the current in vitro study is that human ADSCs stimulated with 5-aza-C were capable of differentiating into myoblasts, and the myodifferentiation was further enhanced by co-stimulation with 5-aza-C and IL-6/sIL-6R. These results imply that ADSCs as well as SCs are important progenitors of myogenic lineage cells. Our results are also supported by the previous finding that murine pulpal stem cells and fetal bovine bone marrow-derived MSCs were induced to differentiate into myoblasts after stimulation with 5-aza-C [9, 31]. Following acute muscle injury, fibro-adipogenic progenitor cells (FAPs) rapidly enter the cell cycle and secrete trophic factors that support the myogenic activity of SCs [32]. Sarcopenia, a condition that has become prevalent in recent years, has been associated with a decrease in the number and function of SCs [11]. Therefore, FAPs, or ADSCs in this context, may serve as an important driver of skeletal muscle regeneration, replacing SCs.

Once activated, SCs differentiate into various cells of the myogenic lineage and eventually form muscle fibers. However, in mice lacking the *IL-6* gene, myogenesis was suppressed compared to wild-type mice [33]. In a mouse model of juvenile muscular dystrophy (mdx mice), IL-6 was upregulated in injured or regenerating muscles as well as skeletal muscles [34]. Based on the above findings, it can be concluded that IL-6 produced by muscles under physiological conditions is a beneficial humoral factor that promotes muscle regeneration. In contrast, in pathological conditions that cause inflammation throughout the body, IL-6 has a harmful effect on muscles. For example, in alive mdx mice, a reduction in IL-6 was associated with attenuated muscular dystrophy, whereas overexpression of IL-6 led to more severe symptoms [35]. Treatment with an IL-6R-neutralizing antibody alleviated symptoms and promoted skeletal muscle regeneration [35]. Patients with rheumatoid arthritis, an autoimmune disease characterized by systemic chronic inflammation, also have elevated levels of circulating IL-6, which promotes sarcopenia [11]. Thus, IL-6 has opposing effects on skeletal muscle in “transient and localized conditions” and “chronic and systemic pathological conditions.” Treatment of murine myoblast-derived C2C12 cells and human primary myoblasts with high concentrations of IL-6 resulted in decreased expression of interleukin-6 receptor (IL-6R) and suppressed cell proliferation 24 h post-treatment, thereby promoting myogenesis [21]. On the other hand, treatment with low concentrations of IL-6 resulted in increased expression of IL-6R and suppressed myogenesis 24 h post-treatment, promoting cell proliferation [21]. These findings suggest that the normal action of IL-6 via membrane-type IL-6R negatively regulates myogenesis. Waldemer–Streyer et al. have proposed that the mechanism of action of TNF-a on the skeletal muscle may differ depending on the physiological or pathological conditions of the body, which may lead to differences in the production of TNF-a and its effects on skeletal muscle [36]. Similar molecular mechanisms may exist for IL-6.

In patients with sarcopenia, ADSCs present in the fat in muscle tissue (ectopic fat) are considered to differentiate into adipocytes, resulting in decreased muscle mass. However, the present in vitro study uncovered the fact that ADSCs differentiate into myoblasts. This is a critical finding that may be reproducible in vivo. Older patients with sarcopenia have decreased the number and function of SCs. If ADSCs localized in fat, which often increases in sarcopenia, can be induced to differentiate into myoblasts, it could lead to the development of a new cell therapy, thus making the current findings highly important. Furthermore, it is useful to utilize unnecessary fat from which a large number of ADSCs can be isolated.

This part of the discussion involves therapeutic strategies for skeletal muscle regeneration using ADSC-based cell therapy. Our study’s results lead us to expect that injecting 5-aza-C and IL-6/sIL-6R into the human skeletal muscle will induce myogenesis through their action on ADSCs. There is also concern that exposure to the inflammatory cytokine IL-6 may cause chronic inflammation, leading to muscle atrophy [37]. Therefore, from the perspective of skeletal muscle regenerative medicine, implanting only IL-6/sIL-6R is not an appropriate strategy. A recent study showed that injecting BMSCs into the mouse skeletal muscle induced muscle hypertrophy [38]. These findings suggest that optimized skeletal muscle regeneration may be achieved without causing muscle fiber atrophy, by stimulating ADSCs with 5-aza-C and IL-6/sIL-6R for unidirectional differentiation into myogenic lineage cells and then transplanting ADSCs by injection. Although there is concern that ADSCs, which are stem cells localized in fat, may differentiate into adipocytes, we have already confirmed through in vitro studies that co-stimulation with 5-aza-C and IL-6/sIL-6R does not affect adipogenesis of ADSCs. This fact also supports the safety of this cell therapy strategy. The present results are expected to lead to the development of an optimized cell transplantation tool for skeletal muscle regenerative medicine in patients experiencing various forms of sarcopenia, including rheumatoid arthritis. Further studies are, therefore, needed to verify the findings.

A limitation of this study is that the sample size of human ADSCs obtained was small. Further evaluation using large sample sizes of ADSCs would be needed to strictly elucidate the role of IL-6 expressed by ADSC cells.

## Conclusions

Stimulation of human ADSCs with 5-aza-C induces myogenesis, which is further promoted by co-stimulation with IL-6/sIL-6R. The high expression of IL-6 in human ADSCs suggests their myogenesis potential; however, the expression of IL-6 decreases as they differentiate. For ADSCs to complete differentiation, it may be necessary to add IL-6 at physiological levels expressed in muscles to promote myogenesis. Thus, the sustained activation of IL-6/JAK2/STAT1 is critical for efficient myogenesis of human ADSCs.

## Supplementary Information

Additional file 1. All full-length images of Western blotting data. Uncropped full-length images of Figs. S2 are shown (DESMIN and β-actin).

Additional file 2. All full-length images of Western blotting data. Uncropped full-length images of Figs. 4A are shown (JAK1, JAK2, JAK3, and β-actin).

Additional file 3. All full-length images of Western blotting data. Uncropped full-length images of Figs. 4B are shown (p-STAT1, t-STAT1, p-STAT3, and t-STAT3).

Additional file 4. All full-length images of Western blotting data. Uncropped full-length images of Figs. 4B are shown (p-STAT5, t-STAT5, and β-actin).

Additional file 5. All full-length images of Western blotting data. Uncropped full-length images of Figs. 4C are shown (p-STAT1, t-STAT1, p-STAT3, and t-STAT3).

Additional file 6. All full-length images of Western blotting data. Uncropped full-length images of Figs. 4C are shown (p-STAT5, t-STAT5, and β-actin).

Additional file 7. All full-length images of PCR data. Uncropped full-length images of Figs. 6B are shown.

Additional file 8: Table S1. Key Resources.

Additional file 9: Table S2. Oligonucleotides used for ChIP-PCR and TaqMan primers for RT-qPCR.

Additional file 10: Table S3. Proteins secreted from human ADSCs.

Additional file 11: Supplementary Figure S1. IL-6 production gradually decreased during the myogenesis process of human ADSCs. After stimulating ADSCs with 5-aza-C, IL-6 production in the culture supernatant was measured using the CBA method on days 0, 7, 14, and 21. Supplementary Figure S2. Myogenic proteins were detected in human myoblast induced by 5-aza-C and human skeletal muscle myoblasts (HSMMs). ADSCs were stimulated with 5-aza-C (10 µM) + IL-6/sIL-6R (10 ng/mL). The protein expression of DESMIN was detected by Western blotting. One lot of ADSCs was used. Supplementary Figure S3. TNF-α and IL-1b did not alter the expression of *MYOG*, *PPARγ*, and *RUNX2* induced by 5-aza-C. (A and B) ADSCs were co-stimulated with 5-aza-C and TNF-α (A) or IL-1β (B) and then measured for *MYOG*, *PPARγ*, and *RUNX2* expressions by RT-qPCR on day 18. Five independent lots of ADSCs were used. Data are expressed as mean ± standard deviation. Student's unpaired two-tailed t-test was used for comparisons between two groups. Supplementary Figure S4. Stimulation of human ADSCs with IL-6/sIL-6R did not induce expression of *MYOG*, *PPARγ*, or *RUNX2*. Human ADSCs were co-stimulated with IL-6/sIL-6R and then measured for *MYOG*,*PPARγ*, and *RUNX2* expressions by RT-qPCR on day 18. Five independent lots of ADSCs were used. Data are expressed as mean ± standard deviation. Student's unpaired two-tailed t-test was used for comparisons between two groups.

## Data Availability

Not applicable.

## Abbreviations

ADSCs: Adipose-derived stem cells
IL: Interleukin
ENA: Epithelial neutrophil activating peptide
GRO: Growth-regulated oncogene
MCP: Monocyte chemoattractant protein
TIMP: Tissue inhibitors of metalloproteinases
TNF: Tumor necrosis factor
NHDF: Normal human dermal fibroblasts
MCF-7: Michigan cancer foundation-7
sIL-6R: Soluble interleukin-6 receptor
IgG: Immunoglobulin G
MyHC: Myosin heavy chain
5-aza-C: 5-Aza-2’-deoxycytidine
VSMC: Vascular smooth muscle cells
RQ: Relative quantification
MYOG: Myogenin
LGALS1: Lectin galactoside-binding soluble 1
PPAR: Peroxisome proliferator-activated receptor
RUNX: Runt-related transcription factor
JAK: Janus kinase
β-actin: Beta-actin
STAT: Signal transducer and activator of transcription
DMEM: Dulbecco’s modified Eagle medium
PBST: Phosphate-buffered saline with Tween
MSCGM: Mesenchymal stem cell growth medium
