# Supplementary figures and images for: Critical roles of IL-6 signaling in myoblast differentiation of human adipose-derived mesenchymal stem cells
Source: Inflamm Regen. 2025 Apr 10;45:9. doi: 10.1186/s41232-025-00373-6 (PMC11983861; doi:10.1186/s41232-025-00373-6)

## Slide 1
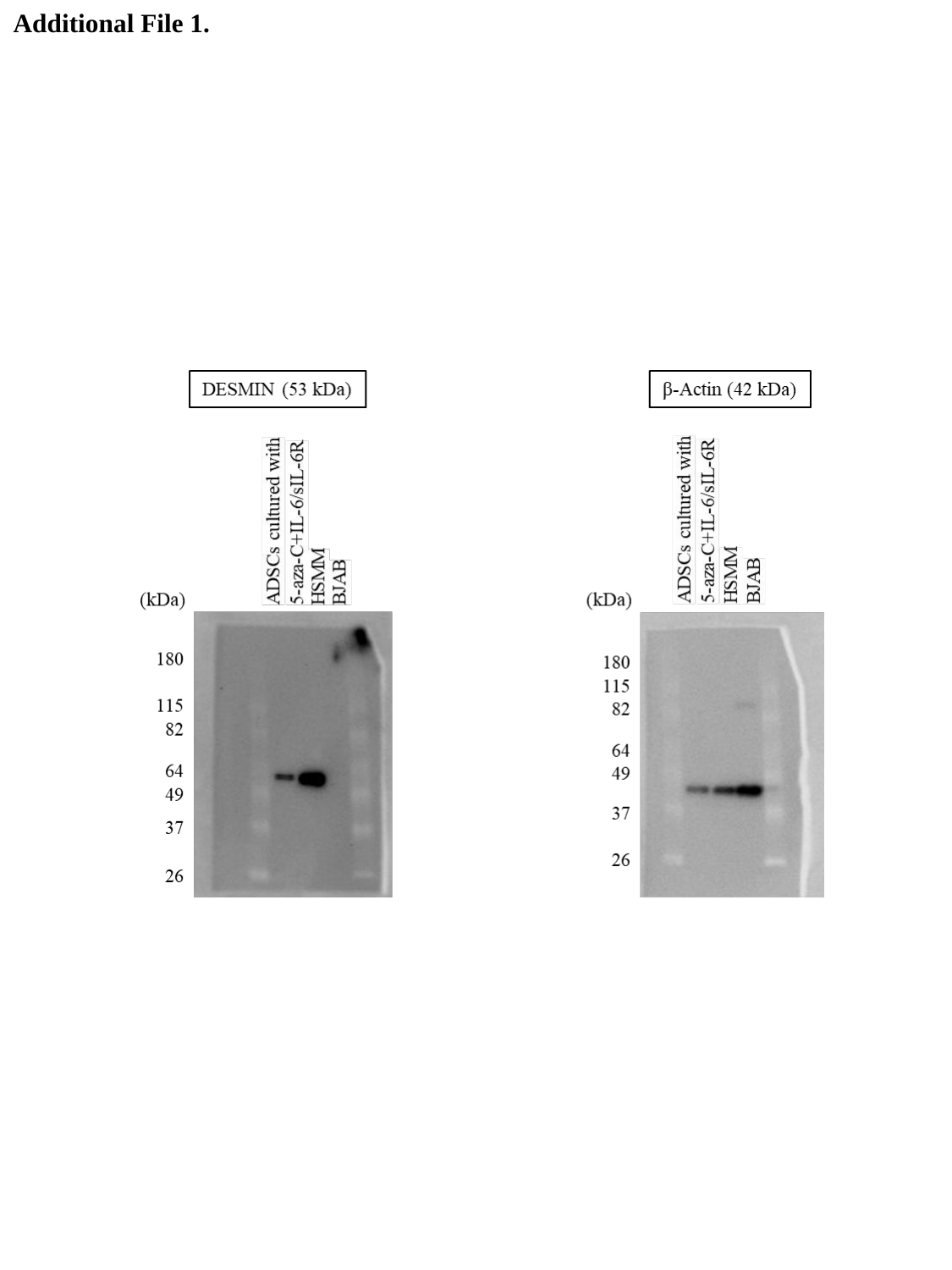

Additional File 1.

Supplement: Supplementary file 1 — Additional file 1. All full-length images of Western blotting data. Uncropped full-length images of Figs. S2 are shown (DESMIN and β-actin). [file 41232_2025_373_MOESM1_ESM.pptx]

## Slide 1
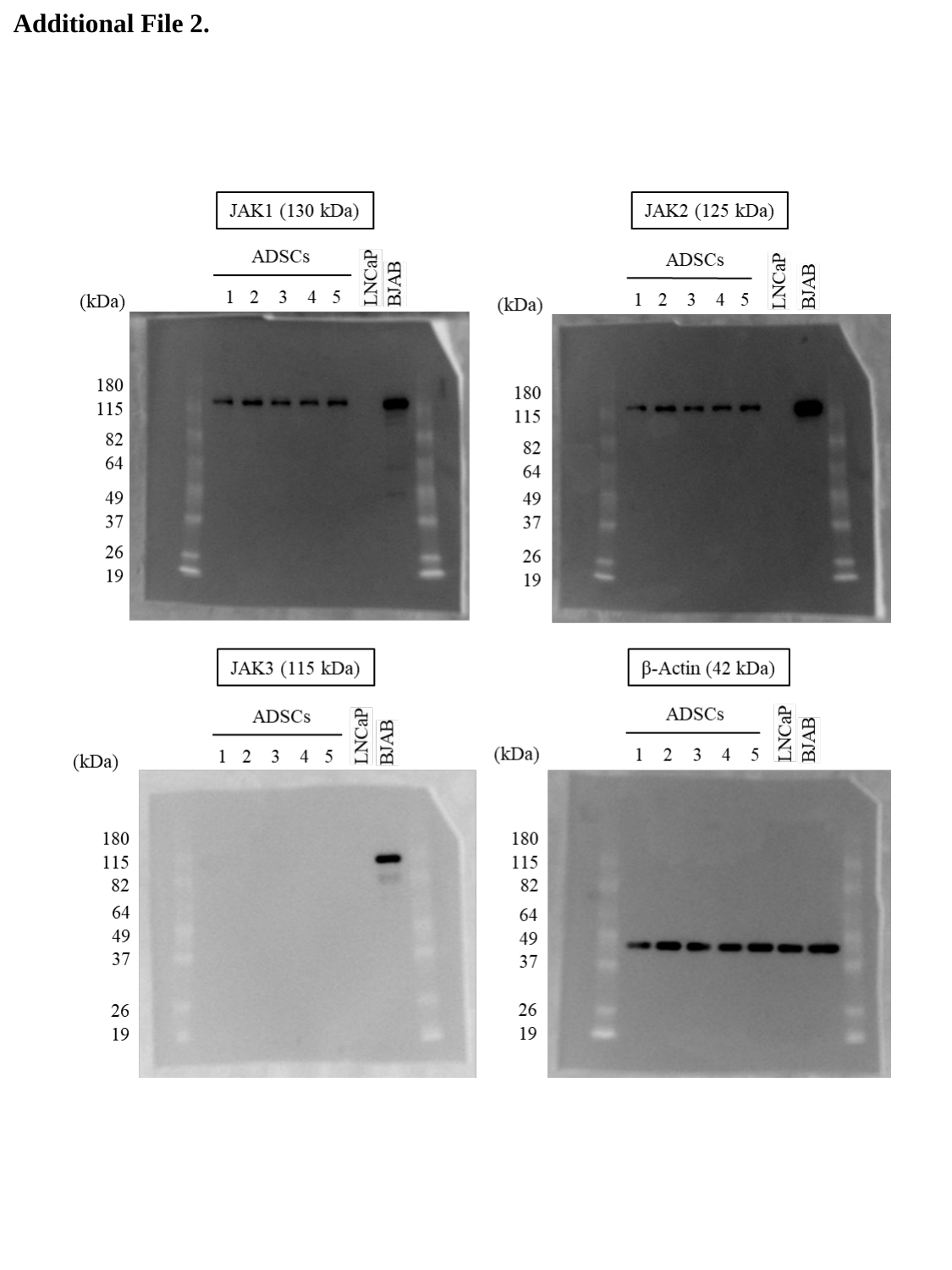

Additional File 2.

Supplement: Supplementary file 2 — Additional file 2. All full-length images of Western blotting data. Uncropped full-length images of Figs. 4A are shown (JAK1, JAK2, JAK3, and β-actin). [file 41232_2025_373_MOESM2_ESM.pptx]

## Slide 1
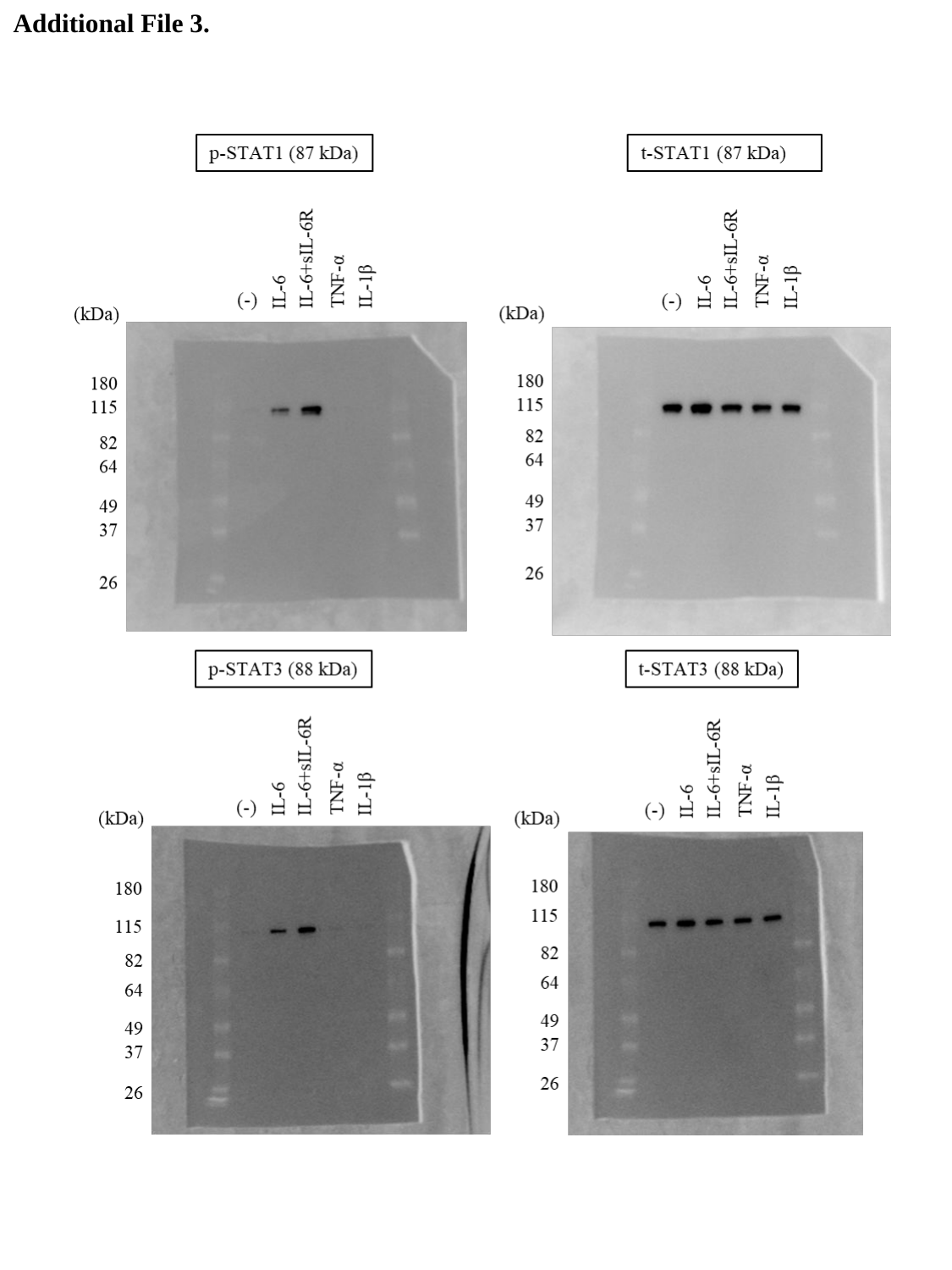

Additional File 3.

Supplement: Supplementary file 3 — Additional file 3. All full-length images of Western blotting data. Uncropped full-length images of Figs. 4B are shown (p-STAT1, t-STAT1, p-STAT3, and t-STAT3). [file 41232_2025_373_MOESM3_ESM.pptx]

## Slide 1
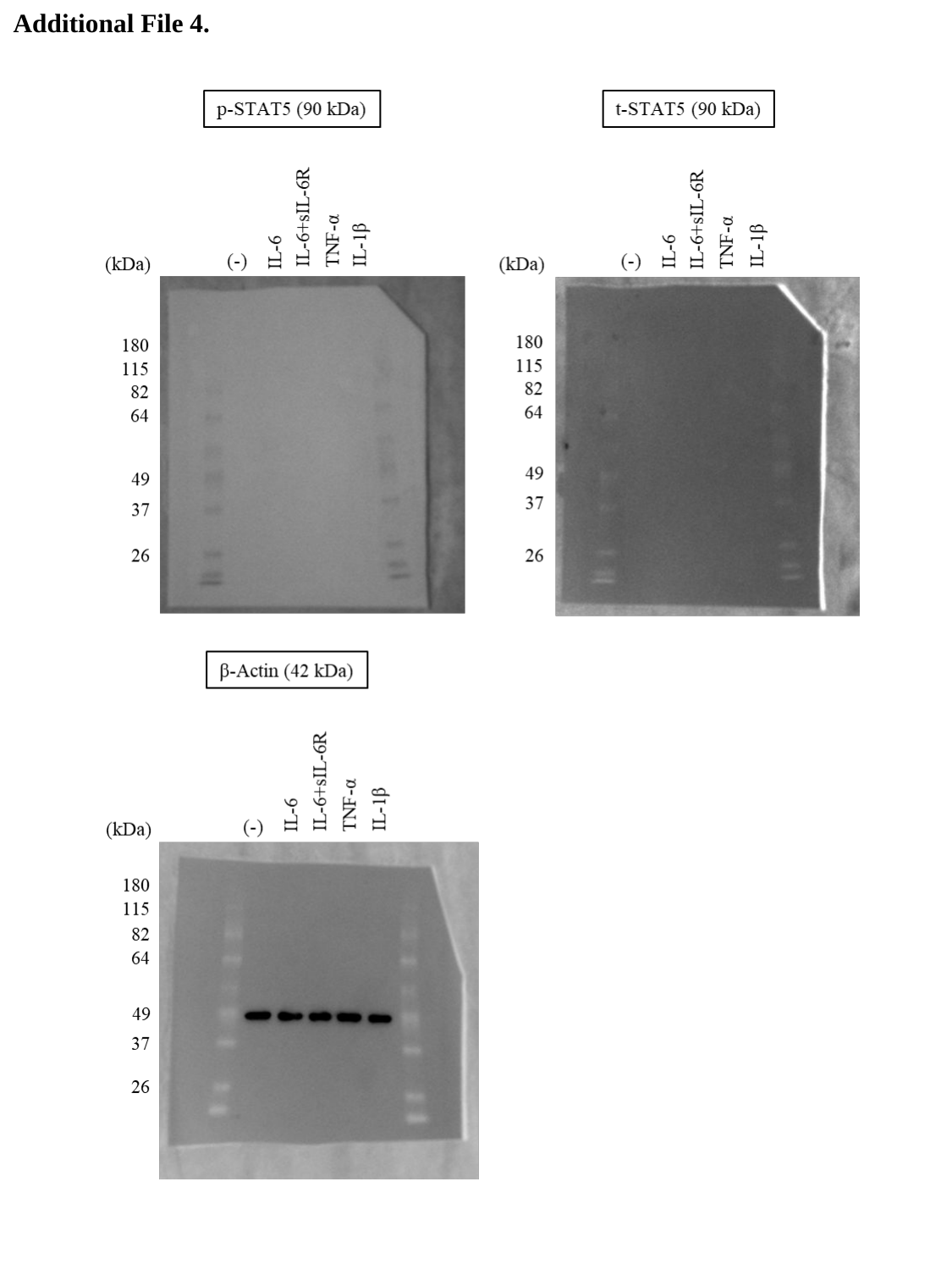

Additional File 4.

Supplement: Supplementary file 4 — Additional file 4. All full-length images of Western blotting data. Uncropped full-length images of Figs. 4B are shown (p-STAT5, t-STAT5, and β-actin). [file 41232_2025_373_MOESM4_ESM.pptx]

## Slide 1
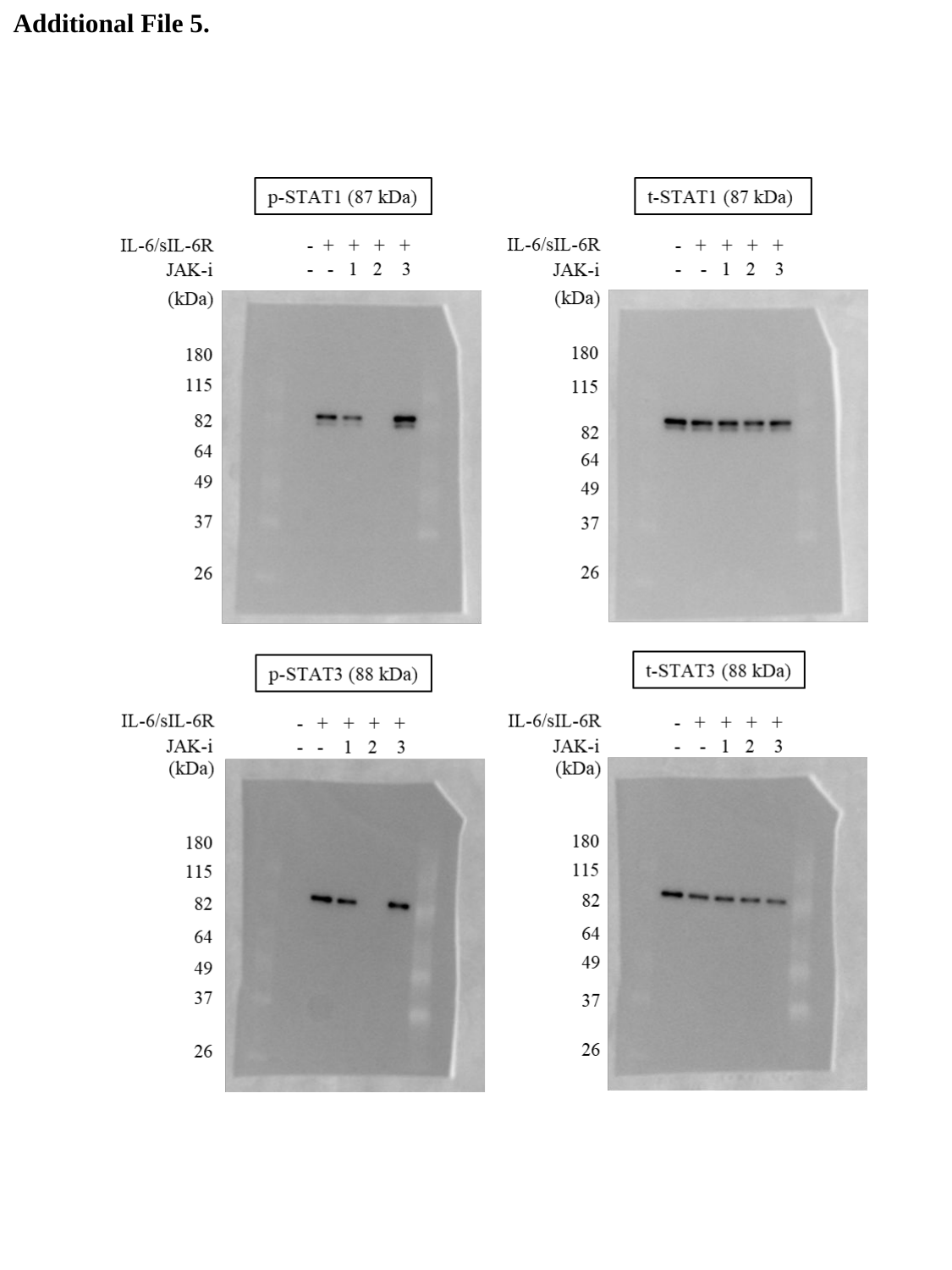

Additional File 5.

Supplement: Supplementary file 5 — Additional file 5. All full-length images of Western blotting data. Uncropped full-length images of Figs. 4C are shown (p-STAT1, t-STAT1, p-STAT3, and t-STAT3). [file 41232_2025_373_MOESM5_ESM.pptx]

## Slide 1
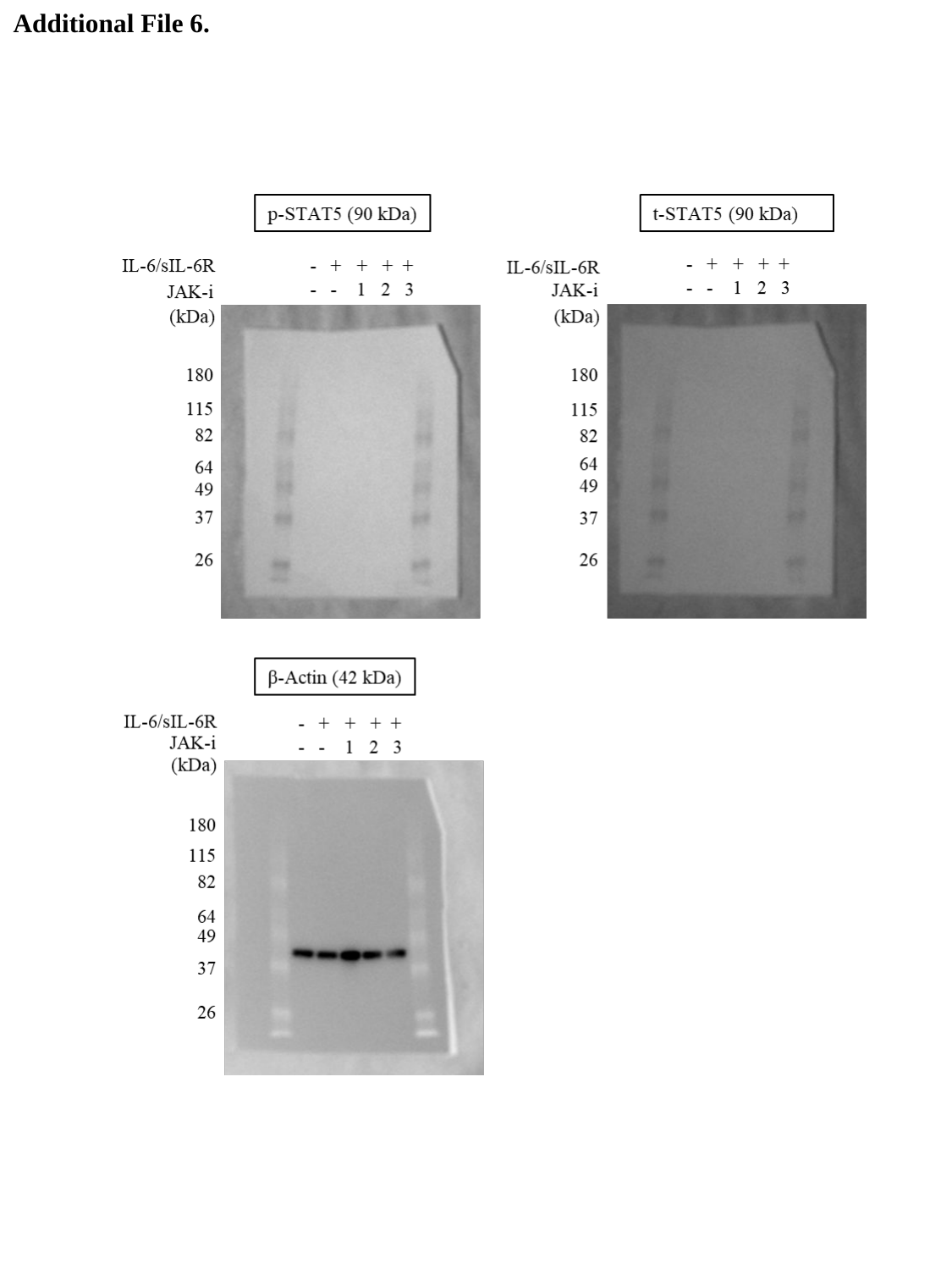

Additional File 6.

Supplement: Supplementary file 6 — Additional file 6. All full-length images of Western blotting data. Uncropped full-length images of Figs. 4C are shown (p-STAT5, t-STAT5, and β-actin). [file 41232_2025_373_MOESM6_ESM.pptx]

## Slide 1
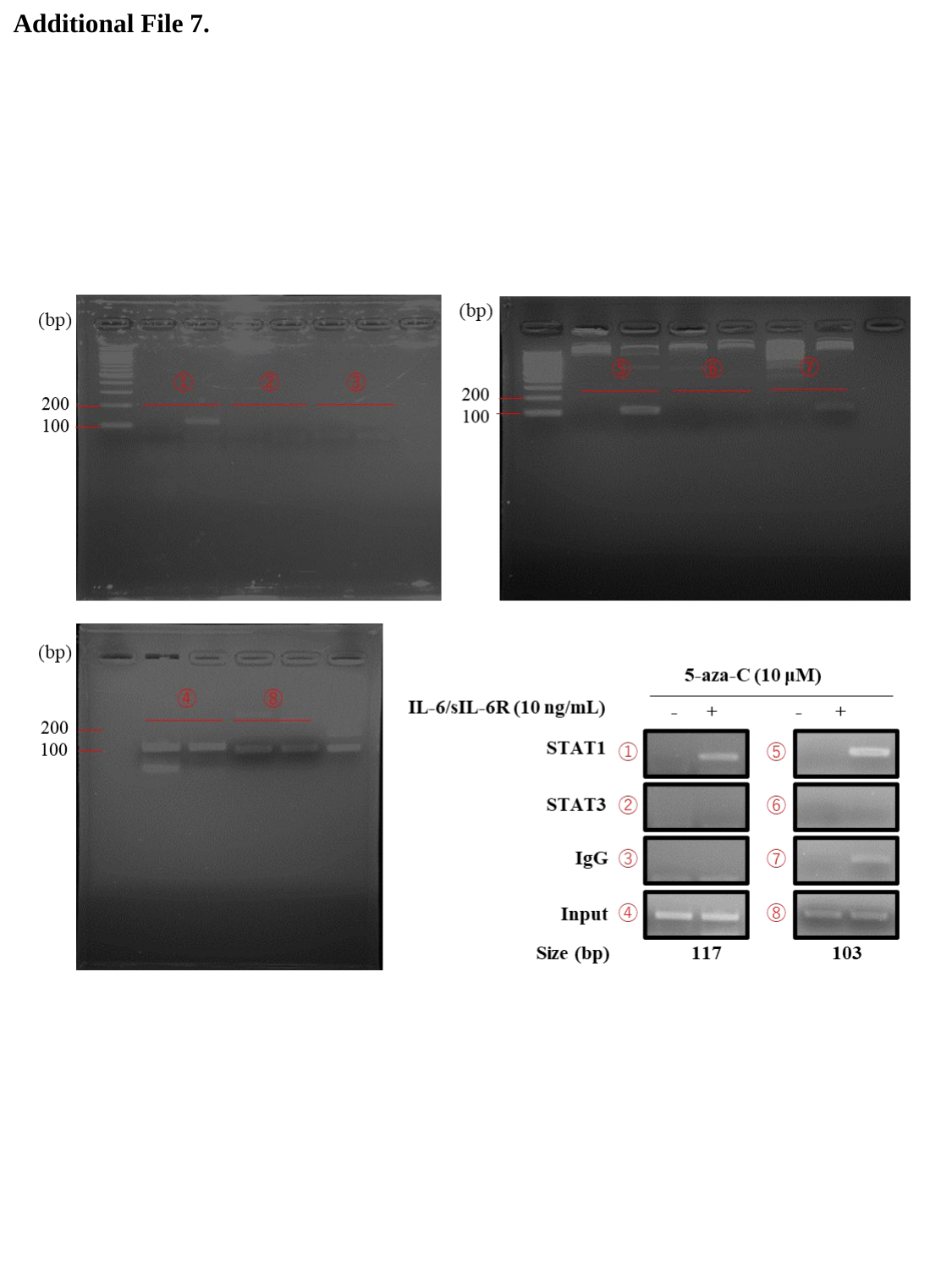

Additional File 7.

Supplement: Supplementary file 7 — Additional file 7. All full-length images of PCR data. Uncropped full-length images of Figs. 6B are shown. [file 41232_2025_373_MOESM7_ESM.pptx]
