# Supplementary material for: Critical roles of IL-6 signaling in myoblast differentiation of human adipose-derived mesenchymal stem cells
Source: Inflamm Regen. 2025 Apr 10;45:9. doi: 10.1186/s41232-025-00373-6 (PMC11983861; doi:10.1186/s41232-025-00373-6)
Supplement: Supplementary file 11 — Additional file 11: Supplementary Figure S1. IL-6 production gradually decreased during the myogenesis process of human ADSCs. After stimulating ADSCs with 5-aza-C, IL-6 production in the culture supernatant was measured using the CBA method on days 0, 7, 14, and 21. Supplementary Figure S2. Myogenic proteins were detected in human myoblast induced by 5-aza-C and human skeletal muscle myoblasts (HSMMs). ADSCs were stimulated with 5-aza-C (10 µM) + IL-6/sIL-6R (10 ng/mL). The protein expression of DESMIN was detected by Western blotting. One lot of ADSCs was used. Supplementary Figure S3. TNF-α and IL-1b did not alter the expression of MYOG, PPARγ, and RUNX2 induced by 5-aza-C. (A and B) ADSCs were co-stimulated with 5-aza-C and TNF-α (A) or IL-1β (B) and then measured for MYOG, PPARγ, and RUNX2 expressions by RT-qPCR on day 18. Five independent lots of ADSCs were used. Data are expressed as mean ± standard deviation. Student's unpaired two-tailed t-test was used for comparisons between two groups. Supplementary Figure S4. Stimulation of human ADSCs with IL-6/sIL-6R did not induce expression of MYOG, PPARγ, or RUNX2. Human ADSCs were co-stimulated with IL-6/sIL-6R and then measured for MYOG,PPARγ, and RUNX2 expressions by RT-qPCR on day 18. Five independent lots of ADSCs were used. Data are expressed as mean ± standard deviation. Student's unpaired two-tailed t-test was used for comparisons between two groups. [file 41232_2025_373_MOESM11_ESM.pptx]

## Slide 1
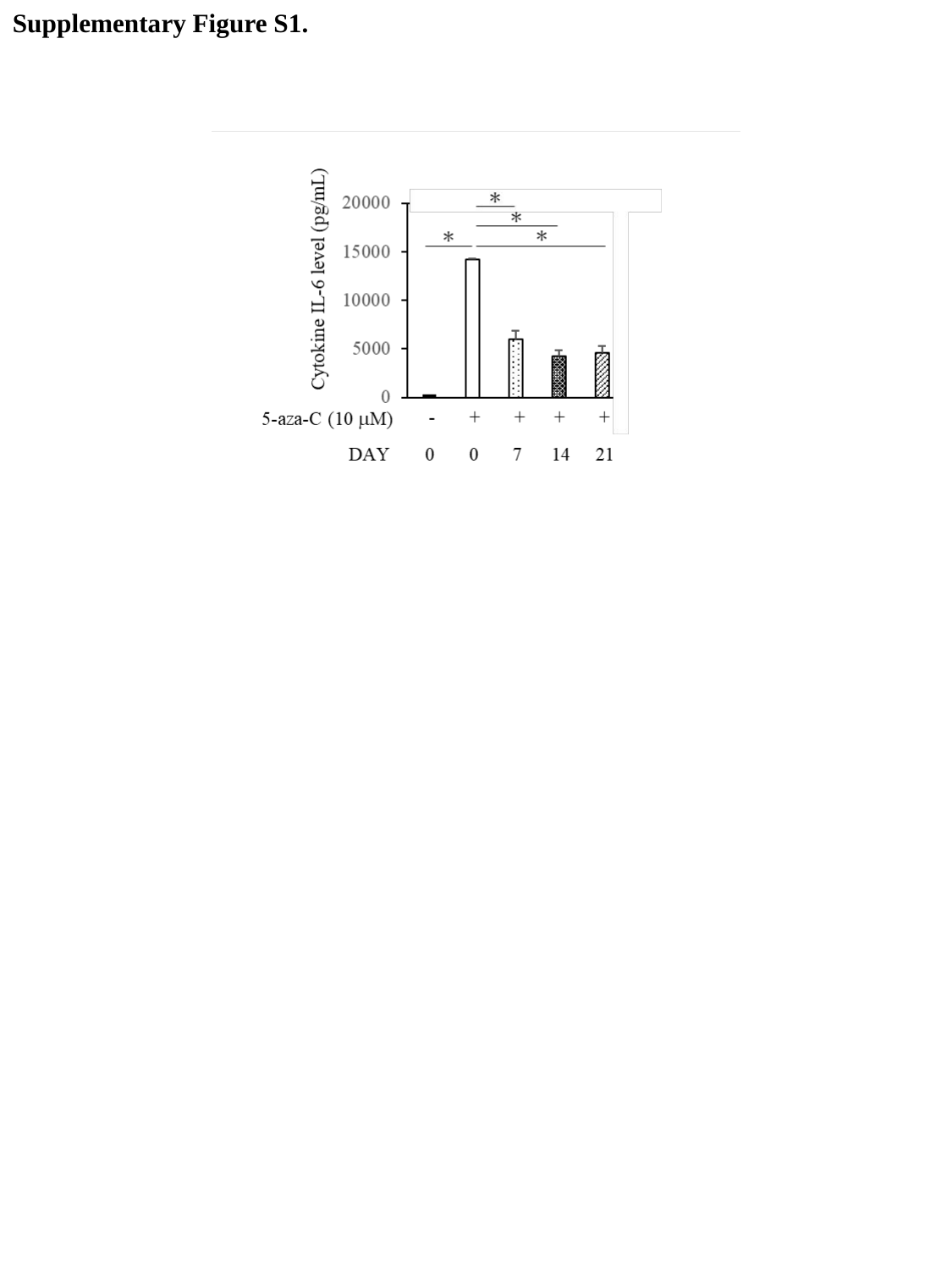

Supplementary Figure S1.

## Slide 2
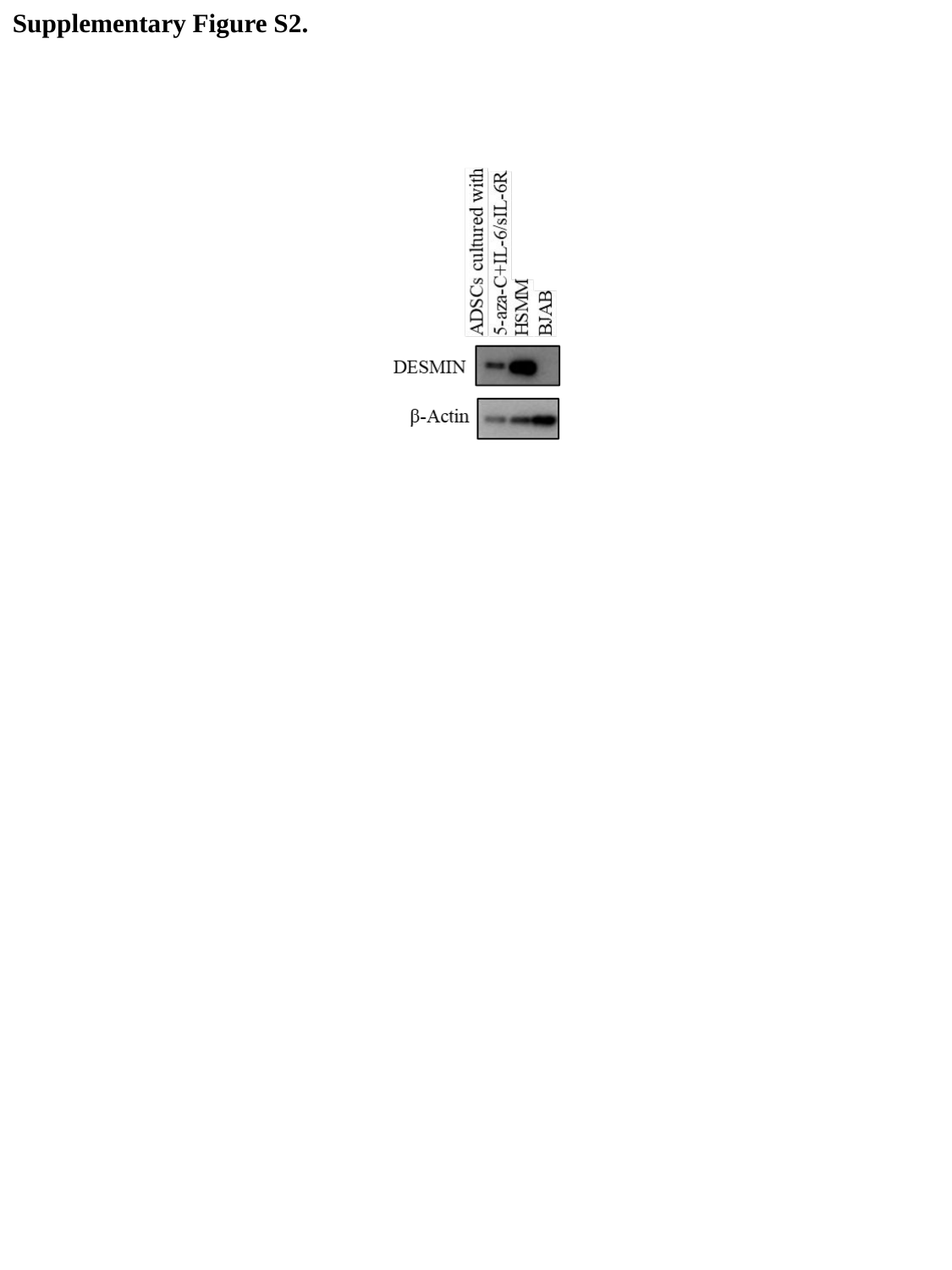

Supplementary Figure S2.

## Slide 3
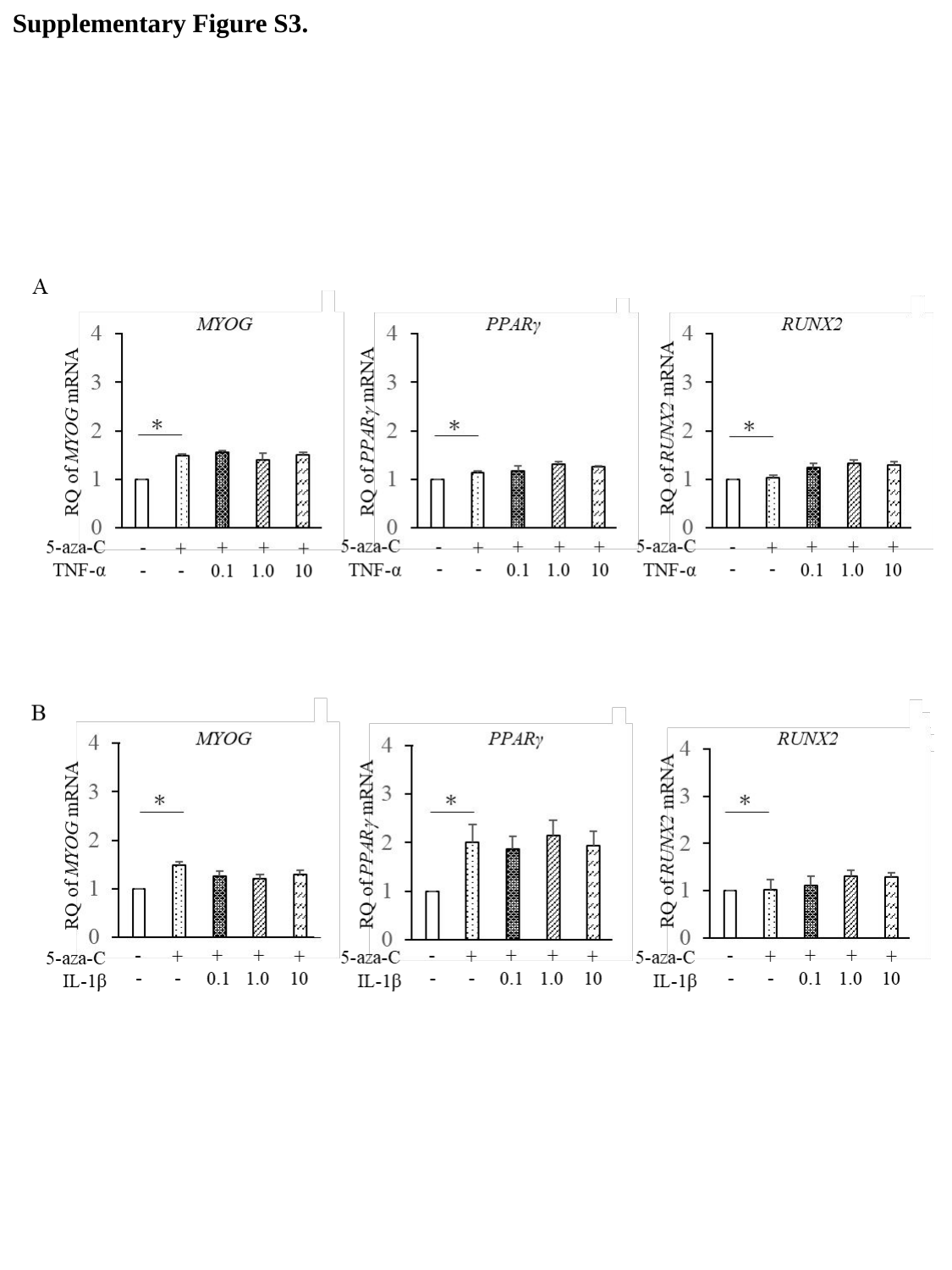

Supplementary Figure S3.

## Slide 4
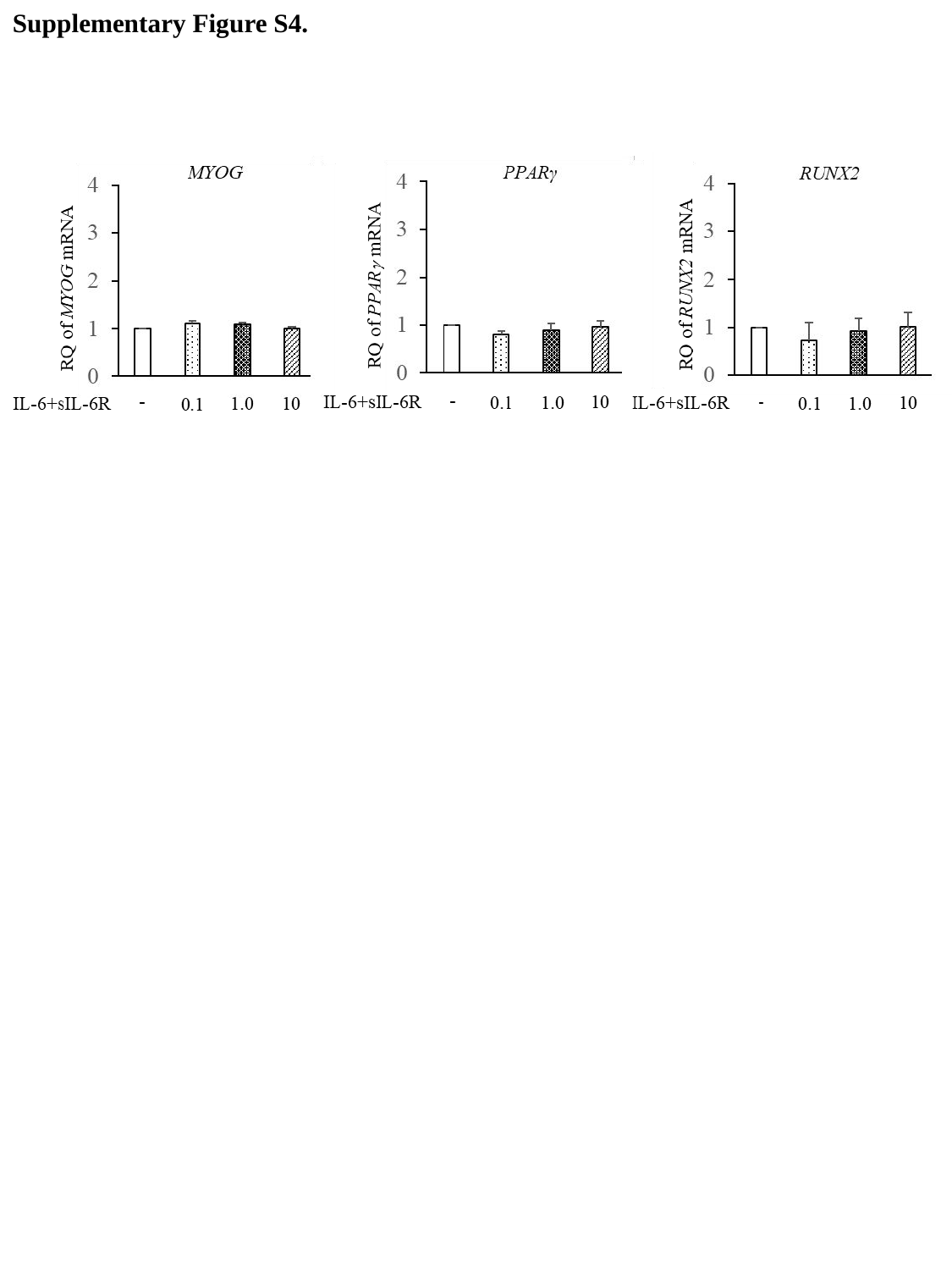

Supplementary Figure S4.
